# Supplementary material for: Content-rich biological network constructed by mining PubMed abstracts
Source: BMC Bioinformatics. 2004 Oct 8;5:147. doi: 10.1186/1471-2105-5-147 (PMC528731; doi:10.1186/1471-2105-5-147)
Supplement: Additional File 2 — The original results of the above study (non-essential files are deleted to keep the file size under the limit set by BMC bioinformatics). [file 1471-2105-5-147-S2.bz2 › chilibotAdditionalFile2/dip30/200ID8652557E689/html/GTF2H1_ERCC5.html]

 


 **GTF2H1** and **ERCC5** 
  
Found 29 abstracts in PubMed, retrieved 29.  
 

 What does Google say? 
 PDF only 
| .edu only 

---

**Interactive relationship** (e.g. stimulation, inhibition, etc)

**Neutral relationship**- Strong functional interactions of TFIIH  [ **GTF2H1** ]  with XPC and XPG  [ **ERCC5** ]  in human DNA nucleotide excision repair, without a preassembled repairosome.  Ref: 11259578 Mol Cell Biol, 2001
- Interactions involving the human RNA polymerase II transcription nucleotide excision repair complex TFIIH  [ **GTF2H1** ] , the nucleotide excision repair protein XPG  [ **ERCC5** ] , and Cockayne syndrome group B CSB protein.  Ref: 8652557 Biochemistry, 1996
- Novel functional interactions between nucleotide excision DNA repair proteins influencing the enzymatic activities of TFIIH  [ **GTF2H1** ] , XPG  [ **ERCC5** ] , and ERCC1 XPF.  Ref: 11141066 Biochemistry, 2001
- XPG  [ **ERCC5** ]  protein interacts with multiple subunits of TFIIH  [ **GTF2H1** ]  and with CSB protein.  Ref: 8652557 Biochemistry, 1996
- At physiological ionic strength, TFIIH  [ **GTF2H1** ]  associated with significant amounts of XPC HR23B and XPG  [ **ERCC5** ]  but not other repair factors.  Ref: 11259578 Mol Cell Biol, 2001
- To identify the forms of TFIIH  [ **GTF2H1** ]  that can function in repair, recombinant XPA, RPA, XPC hHR23B, XPG  [ **ERCC5** ] , and ERCC1 XPF were combined with TFIIH  [ **GTF2H1** ]  fractions purified from HeLa cells.  Ref: 10673506 Genes Dev, 2000
- Furthermore, we reveal that TFIIH  [ **GTF2H1** ]  inhibits the structure specific endonuclease activities of both XPG  [ **ERCC5** ]  and ERCC1 XPF, responsible for the 3 and 5 incision in NER, respectively.  Ref: 11141066 Biochemistry, 2001

**Non-interactive relationship** (e.g. studied together, co-existance, homology, etc.)

- A set of proteins required for repair of most lesions is RPA, XPA, TFIIH  [ **GTF2H1** ] , XPC hHR23B, XPG  [ **ERCC5** ] , and ERCC1 XPF, but additional components have not been excluded.  Ref: 10673506 Genes Dev, 2000
- Human nucleotide excision repair is initiated by six repair factors XPA, RPA, XPC HR23B, TFIIH  [ **GTF2H1** ] , XPF ERCC1, and XPG  [ **ERCC5** ]  which sequentially assemble at sites of DNA damage and effect excision of damage containing oligonucleotides.  Ref: 12138203 Mol Cell Biol, 2002
- Additionally we have examined interactions between TFIIH  [ **GTF2H1** ]  components, the human NER protein XPG  [ **ERCC5** ] , and the CSB protein which is implicated in Cockayne syndrome CS.  Ref: 8652557 Biochemistry, 1996
- Furthermore, reconstitution of the human excision nuclease was observed with a mixture of five repair factors XPA, XPC, XPG  [ **ERCC5** ] , TFIIH  [ **GTF2H1** ] , and RPA and the recombinant XPF ERCC1, thus verifying that no additional protein factors are needed for the specific dual incisions characteristic of human excision repair.  Ref: 9013642 J Biol Chem, 1997
- In mammalian cells, the core factors involved in the damage recognition and incision steps of DNA nucleotide excision repair are XPA, TFIIH  [ **GTF2H1** ]  complex, XPC HR23B, replication protein A RPA, XPG  [ **ERCC5** ] , and ERCC1 XPF.  Ref: 11259578 Mol Cell Biol, 2001
- Core NER enzymes XPA, XPF, XPG  [ **ERCC5** ] , and TFIIH  [ **GTF2H1** ]  are essential for recovery.  Ref: 12531012 DNA Repair Amst , 2002
- For the bulk of mammalian DNA, the core protein factors needed for damage recognition and incision during nucleotide excision repair NER are the XPA protein, the heterotrimeric RPA protein, the 6 to 9 subunit TFIIH  [ **GTF2H1** ] , the XPC hHR23B complex, the XPG  [ **ERCC5** ]  nuclease, and the ERCC1 XPF nuclease.  Ref: 10214908 Biochimie< MedlineTA>Biochimie, 2002
- These results point toward additional roles for TFIIH  [ **GTF2H1** ]  and ATP during NER distinct from a requirement for DNA unwinding in the regulation of the endonuclease activities of XPG  [ **ERCC5** ]  and ERCC1 XPF.  Ref: 11141066 Biochemistry, 2001
- XPA and RPA, once forming a complex at the damage site, would likely work with TFIIH  [ **GTF2H1** ] , XPG  [ **ERCC5** ] , and ERCC1 XPF for dual incision.  Ref: 12486030 J Biol Chem, 2003
- Here, we provide evidence that CSB is found not only in the nucleoplasm but also in the nucleolus within a complex CSB IP 150 that contains RNA pol I, TFIIH  [ **GTF2H1** ] , and XPG  [ **ERCC5** ]  and promotes efficient rRNA synthesis.  Ref: 12419226 Mol Cell, 2002
- The dual incision reaction was reconstituted using the purified mammalian proteins XPA, RPA, XPC, TFIIH  [ **GTF2H1** ] , XPG  [ **ERCC5** ] , and a fraction containing ERCC1 XPF and IF7.  Ref: 8636155 J Biol Chem, 1996
- Stable associations of the ERCC1 complex with other known repair factors XPA, XPC, XPG  [ **ERCC5** ]  and TFIIH  [ **GTF2H1** ]  complex could not be detected.  Ref: 7596355 Mutat Res, 1995
- Finally, damage DNA excision reaction on SV40 minichromosomes was reconstituted with six highly purified NER factors, XPA, XPC HR23B, XPF ERCC1, XPG  [ **ERCC5** ] , RPA and TFIIH  [ **GTF2H1** ] , as those essential for the reaction with naked DNA.  Ref: 10725665 Mutat Res, 2000
- Here we show that with a highly defined system of purified repair factors, DDB can greatly stimulate the excision reaction reconstituted with XPA, RPA, XPC.HR23B, TFIIH  [ **GTF2H1** ] , XPF.ERCC1 and XPG  [ **ERCC5** ] , up to 17 fold for CPDs and approximately 2 fold for 6 4 photoproducts 6 4PPs, indicating that no additional factor is required for the stimulation by DDB.  Ref: 11705987 J Biol Chem, 2002
- The proteins known to be involved in the first steps of the NER reaction damage recognition and incision excision are heterotrimeric RPA, XPA, the 6 to 9 subunit TFIIH  [ **GTF2H1** ] , XPC hHR23B, XPG  [ **ERCC5** ] , and ERCC1 XPF.  Ref: 10526214 Mutat Res, 1999
- The dual incisions are accomplished by sequential and partly overlapping actions of six repair factors, RPA, XPA, XPC, TFIIH  [ **GTF2H1** ] , XPG  [ **ERCC5** ] , and XPF.ERCC1.  Ref: 10373492 J Biol Chem, 1999
- The incision reaction was then reconstituted with the purified proteins RPA, XPA, TFIIH  [ **GTF2H1** ] , XPC, UV DDB, XPG  [ **ERCC5** ] , partially purified ERCC1 XPF complex, and a factor designated IF7.  Ref: 7697716 Cell, 1995
- Transcription coupled repair of 8 oxoguanine requirement for XPG  [ **ERCC5** ] , TFIIH  [ **GTF2H1** ] , and CSB and implications for Cockayne syndrome.Analysis of transcription coupled repair of oxidative lesions here reveals strand specific removal of 8 oxo guanine and thymine glycol both in normal human cells and xeroderma pigmentosum cells defective in nucleotide excision repair.  Ref: 10786832 Cell, 2000
